# Atypical Presentation of Skull Metastasis from Rectal Adenocarcinoma as an Initial Symptom of Recurrence

**DOI:** 10.1155/2012/794354

**Published:** 2012-07-02

**Authors:** Cemal Fırat, Ahmet Hamdi Aytekin, Serkan Erbatur, Nasuhi Engin Aydın, Engin Burak Selcuk

**Affiliations:** ^1^Department of Plastic Surgery, Inonu University School of Medicine, 44380 Malatya, Turkey; ^2^Department of Plastic, Reconstructive and Aesthetic Surgery, Turgut Ozal Medical Center, Inonu University School of Medicine, 44380 Malatya, Turkey; ^3^Department of Pathology, Inonu University School of Medicine, 44380 Malatya, Turkey; ^4^Department of Family Medicine, Inonu University School of Medicine, 44380 Malatya, Turkey

## Abstract

Most malignant rectal tumors are histopathologically characterized as adenocarcinoma and generally metastasize to distant organs such as the lungs or the liver. Metastasis of rectal carcinomas to the skull is extremely rare. This study reports the initial diagnosis of rectal adenocarcinoma recurrence in a 65-year-old female with scalp metastasis. The patient's history indicated a colorectal adenocarcinoma that was resected five years earlier. A skull metastasis from a rectal adenocarcinoma has not yet been reported in the literature as an initial symptom for recurrence. This paper suggests that skull metastasis from any part of the body must be considered in the differential diagnosis of soft tissue tumors in the skull even in the absence of intestinal symptoms.

## 1. Introduction

Cutaneous metastases originating from internal malignancies are uncommon. The frequency of occurrence is about 7% [1, 2]. Although metastatic lesions can occur at any age, their frequency tends to increase in the sixth and seventh decades of life [3, 4]. Breast, lung, and gastrointestinal malignancies are the most likely to metastasize to the skin. However, metastases originating from gastrointestinal malignancies, such as esophageal, gastric, and colorectal tumors, are much less frequent [5, 6]. 

Skin metastases generally appear late in the course of internal malignancies, and often indicate wide-spread neoplasm. Among cutaneous metastases, the skull is a rare site compared with the nodules or alopecia neoplastica commonly detected as clinical signs. It is well-known that breast and lung carcinomas are the most common tumors that cause skull metastasis [7]. We report the unusual case of a patient with a solid nodule on the frontoparietal region of the scalp, which was diagnosed as metastatic adenocarcinoma originating from the rectum. 

## 2. Case Presentation

**Figure 1:**
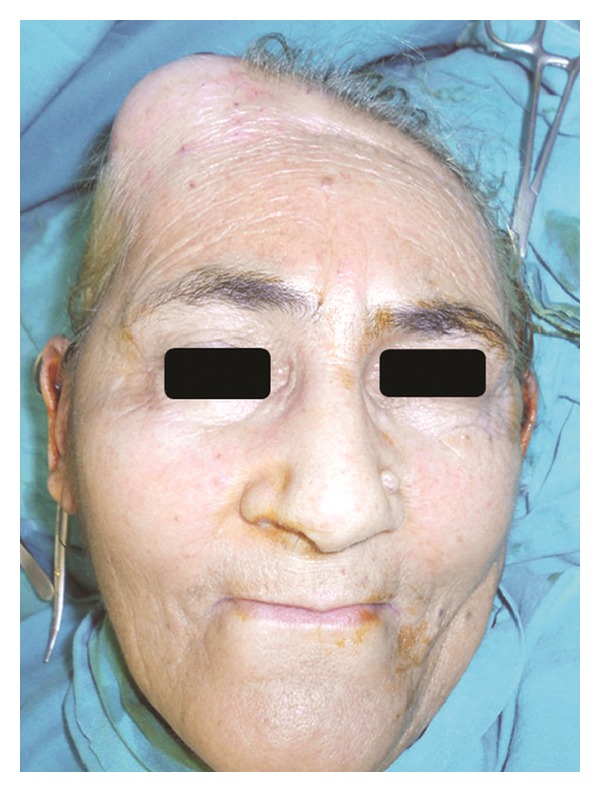
Preoperative view of the patient with a 6 cm diameter mass on the right frontoparietal region.

**Figure 2:**
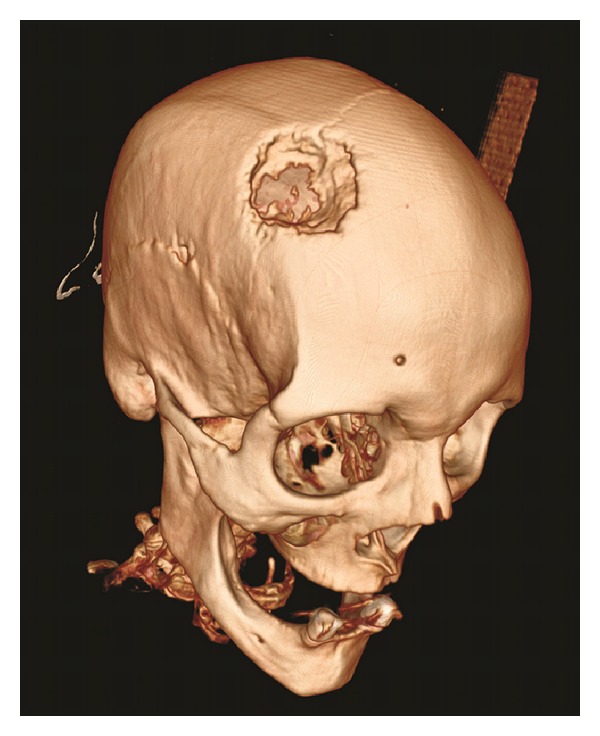
View of the mass in 3D computerized tomography.

A 65-year-old woman presented with a mass 6 cm in diameter located on her right frontoparietal region. The mass had appeared approximately three months earlier and had grown rapidly. The patient's history included resected colorectal adenocarcinoma followed by general surgery and was disease-free for five years. She was referred to us because the swelling and pain indicated a sebaceous cyst or a benign soft tissue tumor (Figure 1). The mass was indurated, round, and adherent to the deep planes. Mild alopecia and skin expansion due to the mass were observed on her anterior scalp. The patient complained of tenderness and mild pain when the mass was palpated. Our clinical differential diagnoses included sebaceous cyst, lipoma, or localized soft tissue tumors. But the plain radiographs and the computerized tomography demonstrated a destructive mass which was eroding the bone over the right frontoparietal region (Figure 2).

**Figure 3:**
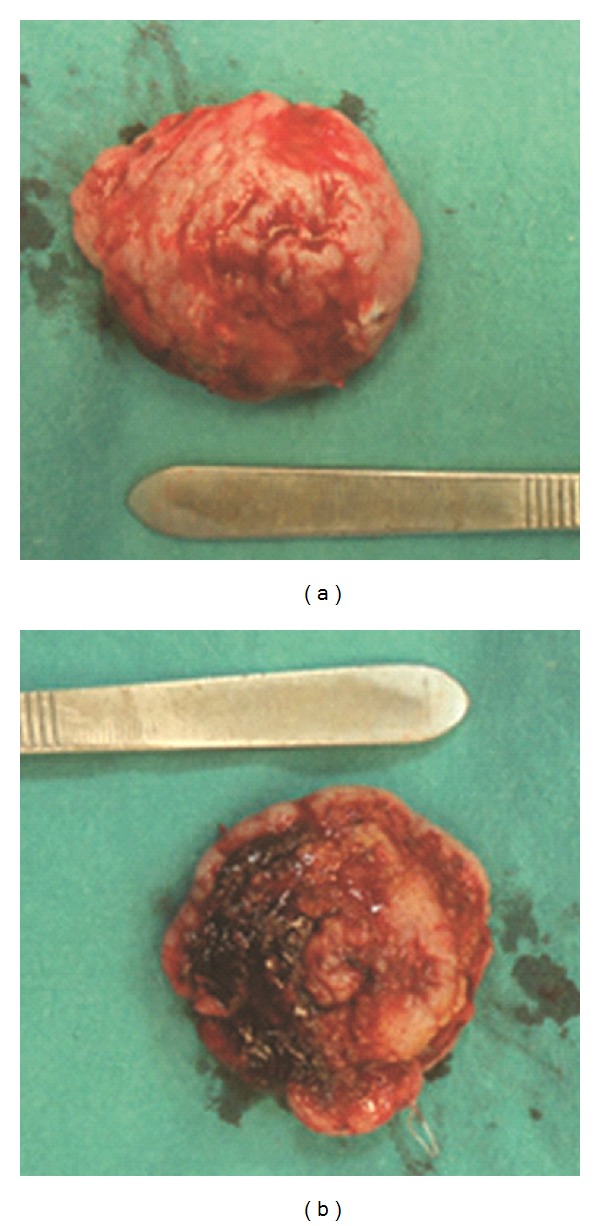
The macroscopic size of mass was measured 5.5 × 5.0 × 2.0 cm.

A transverse incision and a meticulous blunt dissection that were performed under local anesthesia exposed a rubbery soft tissue mass extending to the outer table of the skull. The mass was dissected with care. However, we noticed calvarial bone destruction with an exposed duramater and lytic lesions 2 × 3 cm in size around the calvarial defect. The mass was completely resected. The mass was measured to be 5.5 × 5.0 × 2.0 cm (Figure 3). The damaged bone sites were resected with a 1 cm security margin. A section of the duramater measuring 2 × 3 cm was removed and the defect was reconstructed using a tensor fascia graft by the brain surgeons. Hemostasis was achieved using electrocautery and bone wax. A bolster dressing was applied following the primary closure of the skin incision. The specimen was sent for histopathological analysis. The patient was monitored closely for one day and was discharged the next day.

**Figure 4:**
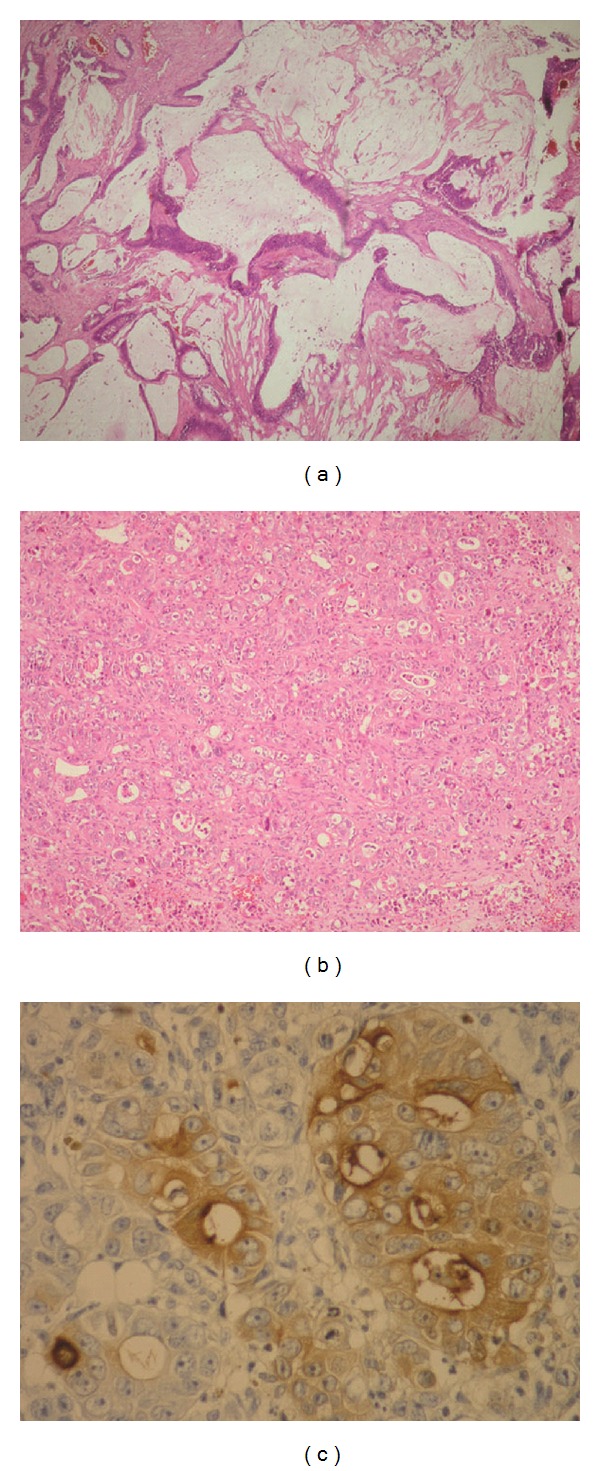
(a) Mucinous adenocarcinoma of the rectum, 5 years ago. (H&E × 200), (b) low differentiated metastatic adenocarcinoma (H&E × 200), (c) immunohistochemical staining with monoclonal CEA positiveness (DAB chromogen × 400).

Histopathologic findings revealed many low differentiated adenoid, and apoptotic and necrotic spaces lined with atypical epithelial islands with vesicular nuclei. At high-power magnification, the specimens showed cytologic atypia with pleomorphic and hyperchromatic nuclei. Immunohistochemical analysis was positive for carcinoembryonic antigen (CEA), villin, and cytokeratins 7 and 20. The specimens were negative for vimentin, chromogranin, thyroglobulin, thyroid transcription factor 1 (TTF-1), and CDX2. The final diagnosis was metastatic low-differentiated adenocarcinoma compatible with the immunophenotype of colorectal adenocarcinoma (Figures 4(a), 4(b), and 4(c)). After the histopathological evaluation revealed metastatic adenocarcinoma, the patient was referred to the medical oncology department.

## 3. Discussion 

Skull metastasis originating from colorectal adenocarcinomas is an unusual site. We presented here a case of metastatic rectal adenocarcinoma to the skull as an initial diagnosis of recurrence. The rectal adenocarcinoma of this patient was diagnosed and treated surgically five years earlier. The asymptomatic interval between the primary resection of the colorectal tumor and the first presentation of scalp metastasis was five years. Scalp metastasis originating from colorectal adenocarcinoma is an uncommon site. To date, only a few metastatic adenocarcinoma from primary gastrointestinal tumors have been reported to migrate to the scalp and to invade the skull [8, 9]. These lesions usually present as firm, rubbery, and asymptomatic subcutaneous nodules. If a detailed examination and radiologic evaluation are not given, these lesions may not be differentiated from soft tissue tumors, granulomas, neurofibromas, or cysts. Histologically, the specimens were negative for CDX2 in different two analyses. Immunohistochemical analysis was positive for carcinoembryonic antigen (CEA), villin, and CK20. Villin is well-known diagnostic marker for colorectal adenocarcinoma [10]. Another focus was not found in the investigations. These results and previous precise diagnosis were evaluated as metastatic colorectal carcinoma.

Colorectal cancer is the second most common type of primary cancer in both men and women in the United States [11]. The most frequent metastatic sites for colorectal adenocarcinoma are the liver, the peritoneum, the pelvis, the lung, and bone [12]. The average time for metastasis to appear is approximately two years after primary tumor resection [11, 12]. Skin metastases are quite rare at the late stages of disseminated tumor, and herald a poor prognosis. However, a skin metastasis can occasionally be the initial presentation or the only sign of an asymptomatic occult neoplasm [3, 13]. Although surgical excision cannot influence the course of the underlying disease, the mean survival time and morbidity can be ameliorated by surgery. In most cases, the abdominal wall appears to be the site preferred by skin metastases [11, 14]. 

In this case, the metastasis originating from rectal adenocarcinoma presented as a recurrence in the skull without any additional metastasis in other organs such as the lung or bones, which are possible pathways for metastasis.

In conclusion, in cases where a rapidly growing mass is encountered on the scalp, metastatic adenocarcinoma originating from an internal malignancy should be taken into consideration as an initial clinical sign of rectal adenocarcinoma recurrence even in the absence of intestinal symptoms.
